# Distinguishing Swine Flu (H1N1) from COVID-19: Clinical, Virological, and Immunological Perspectives

**DOI:** 10.26502/ami.936500125

**Published:** 2023-11-03

**Authors:** Irene Batta, Tejinder Kaur, Devendra K. Agrawal

**Affiliations:** 1 Bothell High School, Bothell, Washington, USA; 2 Department of Zoology, DAV University, Jallandhar, Punjab, India; 3 Department of Translational Research, Western University of Health Sciences, Pomona, California, USA

**Keywords:** Covid-19, Immunity, Influenza A, H1N1 virus, Respiratory Illness, SARS-CoV-2, Swine Flu

## Abstract

This article provides an in-depth examination on the differences between the influenza A strain, H1N1 (also called Swine Flu) and Covid-19 focusing on the immune response and clinical symptoms. Flu symptoms due to influenza A strain, H1N1, were initially discovered in 2009. This variant of influenza A is believed to have emerged through reassortment, a process where the resulting virus inherits gene segments from each of its parental viruses. This reassortment event has resulted in a variant with altered characteristics, potentially affecting the level of immunity in humans. The symptoms of this strain typically manifest 1-4 days after exposure and include fever, cough, sore throat, runny/stuffy nose, body aches, fatigue, and gastrointestinal symptoms such as diarrhea. The transmission dynamics of this new variant, including human-to-human transmission, are still under investigation by health authorities. Individuals with weakened immune systems are generally more susceptible to severe illness. Risk factors associated with swine flu can include older adults, young children, pregnant women, and individuals with obesity. Historical variants of swine flu, such as the 2015 variant in India, have been associated with significant case numbers and deaths, often due to respiratory failure. Since the epidemic of Covid-19 due to SARS-CoV2 in early 2020, several symptoms of COVID-19 and swine flu overlap. In this article, we critically reviewed the differences and similarities in the immune response and clinical symptoms due to H1N1 virus and SARS-CoV2 in human.

## 1. Introduction

Infection with influenza viruses develop symptoms called as Flu. Influenza viruses belong to the orthomyxoviridae family and are classified into four major categories: Influenza A, B, C, and D. Out of these, the former three are known to infect humans while the influenza virus D infects animals, mainly the cattle [1]. Type A and B viruses are known to cause seasonal influenza, commonly known as “flu” [2]. Type C influenza, also known as influenza C, typically tends to induce relatively mild respiratory infections. Type B influenza virus has been found to exhibit similar levels of severity as its counterpart, type A influenza. It is important to note, however, that type B influenza is relatively less prevalent during the flu season when compared to the more dominant type A influenza strain [3]. Influenza A viruses are classified into subtypes based on the combination of their hemagglutinin (HA) and neuraminidase (NA) surface proteins. These subtypes are named using a convention that includes the HA and NA subtype numbers, such as H1N1 or H3N2. Some notable subtypes of influenza A virus are:

(i) H1N1: This subtype is known for causing the 1918 Spanish flu pandemic and has since evolved into seasonal strains. The H1N1 subtype is still included in annual flu vaccines [4].

(ii) H3N2: This subtype is another common seasonal influenza A strain and has caused several outbreaks in the past. It is also included in seasonal flu vaccines [5].

(iii) H5N1: This highly pathogenic avian influenza subtype is often referred to as "bird flu." It primarily infects birds but has caused sporadic and severe human infections, raising concerns about potential pandemics [6].

(iv) H7N9: Another avian influenza subtype, H7N9, emerged in China in 2013. It mainly infects poultry and has resulted in human infections with high mortality rates [7].

(v) H1N2: This subtype is a combination of H1 and N2 and has been found in both humans and pigs [8].

(vi) H2N2: The H2N2 subtype caused the Asian flu pandemic in 1957 but has not been in circulation since 1968 [9].

(vii) H9N2: This subtype is commonly found in poultry and has occasionally infected humans. It is of concern due to its potential to reassort with other subtypes and cause human pandemics [10].

Influenza A H1N1 virus, commonly referred to as H1N1 influenza or "swine flu," that has garnered significant attention due to its potential for both seasonal outbreaks and pandemics. This zoonotic virus is characterized by its genetic diversity, stemming from a combination of avian, swine, and human influenza strains. The most notable event in recent history associated with H1N1 influenza was the 2009 pandemic, caused by a novel reassorted strain that rapidly spread worldwide. H1N1 influenza typically manifests with flu-like symptoms, including fever, cough, sore throat, and fatigue, and can lead to severe respiratory complications [11]. Ongoing surveillance, vaccination efforts, and research on the genetic evolution of H1N1 strains remain critical in our efforts to monitor and control the virus's impact on global public health [12, 13].

Another zoonotic virus, SARS-CoV-2, abbreviated for "Severe Acute Respiratory Syndrome Coronavirus 2," is a novel coronavirus responsible for the ongoing COVID-19 pandemic. This virus was first identified in Wuhan, China, in late 2019 [14] and has since spread globally, resulting in widespread illness and significant public health challenges [15-18]. SARS-CoV-2 is a member of the Coronaviridae family, characterized by its single-stranded RNA genome and spike proteins that enable it to infect human cells by binding to ACE2 receptors. COVID-19, the disease caused by SARS-CoV-2, primarily spreads through respiratory droplets, and is associated with a wide range of symptoms, from mild respiratory issues to severe pneumonia and acute respiratory distress syndrome. Effective public health measures, including vaccination campaigns and non-pharmaceutical interventions, are essential in curbing the transmission of SARS-CoV-2 and managing the impact of the pandemic on global health systems and societies [19-21]. Studying the differences between SARS-CoV-2, the virus responsible for COVID-19, and H1N1 influenza is of paramount significance in the realm of public health and medical research. These two viruses, though both causing respiratory illnesses, exhibit variations in clinical manifestations, transmission dynamics, immunological responses, and pandemic implications. Distinguishing these differences enhances our capacity to manage and respond to outbreaks effectively. It informs medical care, vaccine development, and therapeutic strategies, enabling tailored approaches for each virus. Moreover, understanding the nuances in epidemiology aids in crafting targeted public health measures and resource allocation during pandemics. This comparative analysis also guides global health preparedness efforts, ultimately contributing to our ability to navigate and mitigate the impact of infectious disease threats more comprehensively [22-25].

## 2. Comparative Analysis of Symptoms, Complications, and Transmission Patterns: Influenza H1N1 and COVID-19

Influenza H1N1, commonly known as swine flu, and COVID-19, caused by the SARS-CoV-2 virus, share certain respiratory symptoms, including fever, cough, and fatigue. However, their clinical manifestations and complications differ significantly. Nevertheless, it is imperative to acknowledge that the clinical manifestations and complications exhibited by these viruses diverge substantially from one another. The clinical manifestation of swine flu is characterized by the sudden and rapid onset of symptoms, which may include fever, cough, sore throat, body aches, fatigue, and occasionally gastrointestinal symptoms. In contrast, the symptoms associated with COVID-19, caused by the novel coronavirus SARS-CoV-2, exhibit a wider temporal spectrum, with some individuals experiencing a gradual progression of symptoms over a period of several days. Moreover, a distinctive feature of COVID-19 is the occurrence of asymptomatic cases, wherein infected individuals do not display any discernible clinical signs despite harboring the virus. This phenomenon of asymptomatic carriers poses a significant challenge in effectively identifying and containing the spread of the disease, as these individuals can unknowingly transmit the virus to others, thereby contributing to the overall burden of the pandemic.

The complications associated with swine flu predominantly revolve around respiratory infections, which can further deteriorate pre-existing health conditions or advance to the development of acute respiratory distress syndrome (ARDS). In stark contrast, the novel coronavirus disease (COVID-19) has been found to be intricately linked with an extensive array of complications, encompassing but not limited to, the development of severe pneumonia, acute respiratory distress syndrome (ARDS), intricate vascular disturbances such as the formation of blood clots and the occurrence of strokes, as well as the emergence of multisystem inflammatory syndromes that exert their deleterious effects on both the pediatric and adult populations alike. Furthermore, it is imperative to acknowledge that the ongoing global pandemic caused by the novel coronavirus, COVID-19, has elicited a heightened level of apprehension and unease due to the emergence of a phenomenon known as long COVID. This perplexing condition entails the manifestation of persistent symptoms that extend beyond the acute phase of the illness, thereby engendering a plethora of concerns regarding its potential long-term repercussions on affected individuals [21, 26, 27]. Both viruses primarily spread through respiratory droplets, but COVID-19 may also transmit through aerosols, has a longer incubation period, and exhibits a less predictable seasonality compared to swine flu. Public health strategies, including vaccination campaigns and preventive measures, play crucial roles in controlling their transmission. Understanding the modes of transmission of these viruses is crucial for implementing effective public health measures. While both viruses primarily spread through respiratory routes, they exhibit differences in transmission dynamics, which have important implications for infection control and prevention.

## 3. Transmission Routes of H1N1 Influenza

### 3.1 Respiratory Droplets

The primary mode of transmission for H1N1 influenza is through respiratory droplets expelled when an infected person coughs, sneezes, talks, or breathes. These droplets can carry the virus and infect individuals in proximity, usually within about 6 feet (2 meters) of the infected person. Influenza virus-containing respiratory droplets can land in the mouths or noses of people who are nearby or potentially be inhaled into their lungs, leading to infection [28].

### 3.2 Contact with Contaminated Surfaces

Swine flu can also spread through indirect contact. If a person touches a surface or object contaminated with the virus (e.g., doorknobs, countertops) and then touches their mouth, nose, or eyes, they can become infected. Influenza viruses can survive on surfaces for a limited time, making fomites a potential source of transmission [29].

### 3.3 Hosts Involved

Humans are the primary hosts and transmitters of the H1N1 influenza virus during outbreaks. However, swine (pigs) are the natural hosts of influenza A viruses, including H1N1 strains. Swine flu viruses can circulate among pigs, and sporadically, they can infect humans who have close contact with infected pigs, particularly in agricultural settings. This is known as zoonotic transmission [30].

## 3. Transmission Routes of SARS-CoV-2 (COVID-19)

### 3.1 Respiratory Droplets

Like H1N1 influenza, the primary mode of transmission for SARS-CoV-2 is through respiratory droplets produced when an infected person talks, coughs, sneezes, or breathes. These droplets can contain viral particles and infect individuals who are near the infected person. Respiratory droplets can land in the mouths or noses of nearby people or potentially be inhaled into their lungs, resulting in infection [31].

### 3.2 Airborne Transmission

Unlike H1N1 influenza, there is evidence to suggest that SARS-CoV-2 can be transmitted through smaller respiratory droplets, known as aerosols, which can remain suspended in the air for extended periods. This mode of transmission may occur in enclosed spaces with poor ventilation and prolonged exposure to the virus. Airborne transmission has important implications for indoor settings such as healthcare facilities and crowded spaces [32].

### 3.3 Fecal-Oral Transmission

While not a primary route of transmission, SARS-CoV-2 RNA has been detected in the feces of infected individuals, and there is evidence of potential fecal-oral transmission. This underscores the importance of proper sanitation and hygiene practices, especially in areas with inadequate sanitation facilities [33].

### 3.4 Ocular Transmission

Additional entry point for SARS-CoV-2 could be eyes. Indeed, the SARS-CoV-2 can be found in the ocular secretions of high-viral load patients with active conjunctivitis, where virus could replicate and the vector could be transmitted to several nearby tissues, including conjunctiva, cornea, sclera, and nasolacrimal tissue. There is a strong possibility of the persistence of SARS-CoV-2 in the tear film and could migrate to respiratory and gastrointestinal systems [34].

Experimental evidence suggests that the hosts of Influenza H1N1 (swine flu) and SARS-CoV-2 (the virus responsible for COVID-19) exhibit distinct patterns. In the case of H1N1, pigs are recognized as the natural hosts, and swine flu viruses can circulate among pig populations. However, swine flu viruses have also demonstrated zoonotic potential, occasionally infecting humans in close contact with infected pigs, especially in agricultural settings [35]. Conversely, SARS-CoV-2 primarily infects humans, with experimental studies confirming its ability to infect and replicate in human respiratory cells [36]. While there have been instances of SARS-CoV-2 transmission to certain animals, these cases are generally considered spillover events from humans to animals, and the risk of reverse zoonotic transmission back to humans is under investigation [35]. Experimental data indicate that SARS-CoV-2 has a strong affinity for human ACE2 receptors, facilitating its infection of human cells [17, 26, 36]. These findings emphasize the distinctive host tropism of H1N1 and SARS-CoV-2, with swine flu primarily affecting pigs and COVID-19 predominantly impacting humans.

## 4. Divergent Immunological Responses: H1N1 Influenza vs. SARS-CoV-2 (COVID-19)

The immunological responses triggered by Influenza H1N1 (swine flu) and SARS-CoV-2 (the virus causing COVID-19) are characterized by distinct features and mechanisms. In the case of H1N1 influenza, infection typically leads to the activation of both innate and adaptive immune responses. Upon exposure to the virus, the innate immune system recognizes viral components and initiates the production of proinflammatory cytokines and type I interferons, which serve as early defense mechanisms against viral replication. Subsequently, the adaptive immune system comes into play, with B cells producing antibodies, primarily targeting the viral surface proteins hemagglutinin (HA) and neuraminidase (NA). These antibodies can neutralize the virus, prevent its spread, and confer immunity against specific H1N1 strains. Memory B cells are also generated, allowing for a faster and more robust response upon re-exposure to the virus [37].

On the other hand, SARS-CoV-2 induces a multifaceted immune response characterized by the production of antibodies against various viral proteins, including the spike protein and nucleocapsid protein. Neutralizing antibodies are critical components of the immune response, as they can block viral entry into host cells. However, the immune response to SARS-CoV-2 can exhibit significant variability among individuals. Some individuals may produce high levels of neutralizing antibodies, while others may exhibit delayed or suboptimal antibody responses. In addition to antibody-mediated immunity, T-cell responses play a pivotal role in controlling SARS-CoV-2. CD4+ T cells assist in the activation of B cells and cytotoxic CD8+ T cells, which directly target and eliminate infected cells. This T-cell-mediated immunity not only contributes to viral control but also promotes the formation of immunological memory, offering protection against reinfection [38]. SARS-CoV-2 infection in the gastrointestinal tract and the resultant inflammation could induce changes in gut microbiota and increase anti-microbial agents. This could result in the stimulation of enteric nervous system affecting brain with neurological disorders [39, 40]. However, this warrants further investigation.

## 5. Diagnostic Approaches: H1N1 Influenza vs. SARS-CoV-2 (COVID-19)

The diagnostic approaches for H1N1 influenza and SARS-CoV-2 (the virus responsible for COVID-19) share some similarities but also exhibit distinct differences. Both viruses can be detected using molecular techniques such as polymerase chain reaction (PCR) tests. PCR tests for H1N1 influenza typically target specific genetic sequences of the virus, allowing for accurate identification. Similarly, PCR tests for SARS-CoV-2 detect viral RNA, primarily from respiratory samples like nasopharyngeal swabs, and have been widely used for diagnosing COVID-19 [41]. Additionally, rapid antigen tests are available for both viruses, providing quick results, although they may have lower sensitivity compared to PCR tests.

However, differences emerge in terms of serological testing. While serological tests such as enzyme-linked immunosorbent assays (ELISA) are commonly used to detect antibodies against SARS-CoV-2 in blood samples, they are less commonly employed for H1N1 diagnosis. Serological tests can indicate past infection or the presence of antibodies due to vaccination, offering valuable information for COVID-19 surveillance and understanding population immunity [42]. In contrast, serological testing plays a limited role in H1N1 diagnosis. In summary, while both H1N1 influenza and SARS-CoV-2 can be diagnosed using PCR and rapid antigen tests, the inclusion of serological testing is more prominent in the diagnosis of COVID-19, providing insights into past infections and vaccination status.

## 6. Vaccine Strategies for H1N1 Influenza and COVID-19

The vaccines for H1N1 influenza and SARS-CoV-2 (COVID-19) share common principles of inducing immunity but differ in several aspects. In the case of H1N1 influenza, seasonal flu vaccines are developed each year to target specific strains of the virus based on surveillance data [1]. These vaccines contain inactivated or attenuated influenza viruses, and individuals are encouraged to receive a new vaccine each flu season. In contrast, COVID-19 vaccines, such as those developed by Pfizer-BioNTech, Moderna, Johnson & Johnson, and others, primarily focus on the spike protein of SARS-CoV-2. They use innovative mRNA or viral vector technologies to trigger an immune response [43-45]. COVID-19 vaccines have played a pivotal role in the global response to the pandemic and have been authorized for emergency use to control the spread of the virus. Continuous monitoring and adaptation of both vaccine strategies are essential as new variants of the viruses may impact vaccine effectiveness.

## 7. Treatment Approaches for H1N1 Influenza and COVID-19

The treatment approaches for H1N1 influenza and SARS-CoV-2 (the virus responsible for COVID-19) involve both similarities and differences. Antiviral medications play a crucial role in managing both infections. For H1N1 influenza, antiviral drugs like oseltamivir (Tamiflu) and zanamivir (Relenza) are effective when administered early in the course of the illness [46]. These drugs inhibit the replication of the influenza virus and can reduce the severity and duration of symptoms. Similarly, for COVID-19, antiviral therapies like remdesivir have been developed and authorized for use in certain cases [47]. These drugs target the replication of SARS-CoV-2 and have shown benefits in hospitalized patients with severe disease. However, differences arise in the use of other treatments. In the case of COVID-19, the use of corticosteroids such as dexamethasone has been found to reduce mortality in severe cases [48]. These medications help manage the excessive immune response and inflammation often seen in severe COVID-19. Additionally, monoclonal antibody therapies have been authorized for COVID-19 treatment, offering passive immunity, and reducing the risk of severe disease progression [49]. However, adverse hematological events associated with COVID-19 therapeutics and vaccination have been noted, and must be carefully examined, particularly in patients with hematological malignancies [50, 51].

In contrast, the treatment of H1N1 influenza does not commonly involve corticosteroids or monoclonal antibodies. Supportive care, including fever reducers and hydration, is a mainstay of treatment for both infections. Vaccination is a critical preventive measure for both H1N1 influenza and COVID-19. In summary, while antiviral drugs are central to the treatment of both H1N1 influenza and COVID-19, differences exist in the use of corticosteroids and monoclonal antibodies, reflecting variations in the immunopathology of these viral infections.

## 8. Previous Swine Flu Variants and Patterns

Swine flu, caused by various strains of the influenza A(H1N1) virus, has exhibited different patterns and impacts over the years. Here, we discuss three notable periods and their associated flu patterns:

### 8.1 2009 Global Pandemic

The 2009 H1N1 influenza pandemic stands as a significant milestone in the history of infectious diseases, exhibiting a distinctive profile compared to routine seasonal influenza outbreaks. This pandemic, caused by a novel reassorted strain of the H1N1 influenza virus, left an indelible mark on global public health. It is essential to delve deeper into the details of this pandemic to comprehend its unique characteristics and the lessons it offered for future preparedness.

Epidemiological studies estimated a staggering 60.8 million cases of H1N1 influenza globally during the pandemic period, underscoring its remarkable contagiousness and capacity to rapidly spread across borders [52]. The morbidity and mortality associated with this strain were substantial, with an estimated 284,400 deaths attributed to H1N1 influenza [52]. These figures accentuate the pandemic's gravity and its impact on public health systems worldwide.

What sets the 2009 H1N1 pandemic apart from typical influenza outbreaks is its occurrence during both the northern hemisphere's summer months and the more expected cooler months [53]. Seasonal influenza, in contrast, typically peaks during the colder seasons. This unique seasonality challenged public health authorities and posed questions about the virus's behavior and transmission dynamics.

Comprehensive surveillance and robust response strategies proved imperative during the 2009 H1N1 pandemic. The World Health Organization (WHO) declared it a public health emergency of international concern [54], highlighting the need for international cooperation and information sharing to address the rapidly evolving situation. Surveillance systems, such as the Global Influenza Surveillance and Response System (GISRS), played a crucial role in monitoring the virus's spread and evolution [12]. Antiviral medications, like oseltamivir and zanamivir, were used to mitigate the illness's severity [52]. Moreover, vaccination campaigns were initiated to curb the virus's transmission and reduce its impact on vulnerable populations [55].

### 8.2 2015 Outbreak in India

The resurgence of H1N1 influenza in India in 2015 marked a concerning public health event, with a notable increase in both the number of cases and deaths compared to the initial outbreak in 2009. By March 15, 2015, the country had reported nearly 30,000 confirmed cases and over 1,700 deaths, surpassing the figures observed during the 2009 outbreak (27,236 cases and 981 deaths [56]. Several factors contributed to this resurgence and the heightened impact of the H1N1 variant in India. One significant factor was the gaps in population immunity. While the 2009 pandemic had exposed a substantial portion of the population to the H1N1 virus, immunity levels may have waned over time. This decrease in immunity could leave a larger segment of the population susceptible to the virus's resurgence, potentially leading to a higher number of cases and more severe outcomes [57].

Climatic conditions also played a role in the resurgence. Influenza viruses often exhibit seasonal patterns, with increased transmission during cooler and drier months. India's climatic conditions, including cooler temperatures in 2015, might have created a more favorable environment for the virus to spread. The influenza virus can remain stable and infectious for longer periods in cold and dry conditions, potentially contributing to increased transmission [58].

Public health measures, such as vaccination campaigns and surveillance, played a critical role in responding to the resurgence of H1N1 in India. The government and healthcare authorities-initiated vaccination drives to protect vulnerable populations and mitigate the spread of the virus. Additionally, enhanced surveillance and early detection efforts helped in identifying cases promptly and implementing control measures.

### 8.3 2023 Case and Transmission

The case of the 42-year-old patient in June 2023, who tested positive for H1N1 despite having no direct contact with pigs, raises intriguing questions about the transmission patterns and individual susceptibility to the virus. The patient's presentation with symptoms like fever, headache, sore throat, and abdominal pain underscores the clinical variability of H1N1 influenza, which can manifest with a range of symptoms, from mild to severe. Tragically, her illness resulted in a fatal outcome on May 5, 2023, emphasizing the potential seriousness of H1N1 infections, particularly in individuals with underlying medical conditions [59]. One striking aspect of this case is that two of the patient's close contacts who did have contact with pigs did not test positive for H1N1 and did not develop any respiratory illness. This observation highlights the complexity of H1N1 transmission dynamics. While H1N1 is primarily considered a zoonotic virus that can transmit between pigs and humans, human-to-human transmission is often the predominant mode, especially during outbreaks and pandemics. The fact that individuals with direct pig contact did not contract the virus suggests that other factors, such as viral strains, viral load, and individual immunity, may influence susceptibility to H1N1 [30]. This case serves as a reminder of the need for ongoing surveillance and research into the transmission patterns and factors that contribute to the variability in H1N1 infections. Understanding the dynamics of H1N1 transmission is crucial for developing effective prevention and control strategies, as well as for improving our preparedness for potential outbreaks of this influenza subtype.

## 9. Previous COVID-19 Variants and Patterns

### 9.1 2002-2003 SARS

The Severe Acute Respiratory Syndrome (SARS) outbreak in 2002-2003 marked a significant event in the realm of respiratory viral infections. SARS is caused by a coronavirus known as SARS-CoV and belongs to the same virus family as the current COVID-19-causing virus, SARS-CoV-2. The SARS outbreak first emerged in November 2002, and within a remarkably short span of just 11 weeks, it had spread to 27 countries, demonstrating its potential for rapid global transmission [60]. During the SARS outbreak, approximately 8,096 cases were reported worldwide, with a concerning fatality rate of 774 deaths. These numbers underscored the severity of the disease and the significant impact it had on affected individuals and healthcare systems. The SARS outbreak was characterized by symptoms such as high fever, cough, and severe respiratory distress, which often led to pneumonia [61]. SARS-CoV was primarily transmitted from person to person through respiratory droplets, and the virus's ability to spread rapidly highlighted the importance of effective public health measures and international cooperation in controlling such outbreaks. The experience with SARS served as a valuable lesson for preparedness and response to future emerging respiratory viruses, including the subsequent emergence of COVID-19 in late 2019 [62].

### 9.2 2019-2023 COVID-19

The emergence of COVID-19 in late 2019 marked the beginning of a global health crisis. The virus responsible for COVID-19, known as SARS-CoV-2, was initially identified in Wuhan, China, and its early connections were traced to the Huanan Seafood Wholesale Market, where live animals were also sold [63]. This market was considered a potential source of zoonotic transmission from animals to humans, although the exact origins of the virus are still a subject of ongoing investigation. As cases of COVID-19 began to be confirmed not only in Wuhan but also in other parts of China and subsequently in various countries around the world, international efforts were initiated to contain its spread. Screening measures were implemented for flights originating from Wuhan, with the aim of identifying and isolating potential cases in other countries [64]. However, the highly contagious nature of SARS-CoV-2 led to person-to-person transmission, causing the virus to spread rapidly. To mitigate the spread of the virus, many countries and regions implemented strict public health measures, including lockdowns, quarantines, travel restrictions, mask mandates, and social distancing requirements [65]. These containment measures were crucial in slowing the transmission of the virus and buying time for healthcare systems to prepare for the influx of COVID-19 cases. The global response to COVID-19 has involved coordinated efforts by governments, healthcare organizations, scientists, and public health experts. It has included the development and distribution of vaccines, widespread testing and contact tracing, the promotion of mask-wearing and hand hygiene, and ongoing research to understand the virus and its variants better.

### 9.3 Other variants

The COVID-19 pandemic has seen the emergence of several variants of the SARS-CoV-2 virus, each with its unique genetic changes and characteristics. These variants have played a significant role in shaping the course of the pandemic. Notably, the Alpha variant (B.1.1.7), which was first identified in the UK, exhibited enhanced transmissibility, leading to its prevalence in the region [66]. The Beta variant (B.1.351), originating in South Africa, carried multiple spike protein mutations, which allowed it to spread more easily and, concerning public health efforts, demonstrated reduced susceptibility to certain treatments and post-vaccination immunity [67]. In Brazil, the Gamma variant (P.1) was identified, similar to the Beta variant with various spike mutations. Gamma exhibited a concerning pattern of reduced response to treatments and post-infection immunity [68]. Meanwhile, the Delta variant (B.1.617.2), first detected in India, rapidly became the dominant strain in the US and worldwide. It demonstrated heightened transmissibility and was associated with an increased risk of hospitalization [69]. The most recent variant, Omicron (B.1.1.529), identified in South Africa, stands out due to its extensive spike protein changes and other mutations. It is considered highly infectious and has led to concerns about vaccine efficacy, with only certain monoclonal antibody therapies demonstrating effectiveness against it [70]. These variants' emergence and spread underscore the virus's adaptability and the need for continued vigilance in monitoring and responding to new strains. The patterns observed in these COVID-19 variants highlight the virus's diverse impacts on populations worldwide. Factors such as population immunity, climatic conditions, and individual health status play pivotal roles in shaping the severity and spread of the disease. Additionally, the evolving nature of the virus, with changes in spike proteins and potential resistance to treatments, underscores the complexity of managing and controlling COVID-19 on a global scale [71].

## 10. Comparative Analysis of previous Variants and Patterns: Influenza H1N1 and COVID-19

Both previous swine flu variants and COVID-19 variants exhibit similarities in their impact on global health. Both viruses have caused significant outbreaks with varying patterns of illness and death. Factors such as population immunity, climatic conditions, and individual health status have been identified as influential factors in shaping the severity and spread of both diseases. Additionally, the emergence of different variants in both cases has introduced new challenges, with mutations in specific viral components affecting transmission and potential treatment resistance.

Both H1N1 influenza (swine flu) and SARS-CoV-2 (COVID-19) have seen the emergence of various variants, albeit with some notable differences. H1N1 variants have continued to circulate since the 2009 pandemic, and these variants have shown changes in viral components, impacting their transmission and potential resistance to antiviral treatments [72]. In contrast, SARS-CoV-2 variants have arisen more rapidly and have garnered significant attention. Variants such as Alpha, Beta, Gamma, Delta, and Omicron have exhibited unique mutations in the spike protein, contributing to differences in transmission dynamics, disease severity, and responses to treatments and vaccines [73, 74]. While both types of viruses have evolved over time, SARS-CoV-2 variants have posed specific challenges due to their rapid diversification and potential impact on vaccine effectiveness.

## 11. Conclusion

Both previous swine flu variants (influenza A(H1N1)) and COVID-19 have significantly impacted global health, with varying patterns of outbreaks, illness, and death. Factors like population immunity, climatic conditions, and individual health status influence the severity and spread of both diseases. The emergence of different variants in both cases introduces new challenges, with mutations affecting transmission and potential resistance to treatments. While treatment approaches involve supportive care and antiviral medications, COVID-19 has additional treatment options, such as monoclonal antibodies and corticosteroids. Understanding the similarities and differences between these viral diseases is crucial for developing effective prevention, surveillance, and treatment strategies to mitigate their impacts on public health. Ongoing research and global cooperation are necessary to combat these respiratory viruses and ensure the well-being of individuals and communities worldwide.

## Data Availability

Not applicable since the information is gathered from published articles.
